# Blended Learning Compared to Traditional Learning in Medical Education: Systematic Review and Meta-Analysis

**DOI:** 10.2196/16504

**Published:** 2020-08-10

**Authors:** Alexandre Vallée, Jacques Blacher, Alain Cariou, Emmanuel Sorbets

**Affiliations:** 1 Diagnosis and Therapeutic Center, Hypertension and Cardiovascular Prevention Unit Hôtel-Dieu Hospital, Assistance Publique–Hôpitaux de Paris Paris-Descartes University Paris France; 2 Cochin University Hospital Paris France

**Keywords:** blended learning, virtual patients, online learning, computer-aided instruction, traditional learning, meta-analysis

## Abstract

**Background:**

Blended learning, which combines face-to-face learning and e-learning, has grown rapidly to be commonly used in education. Nevertheless, the effectiveness of this learning approach has not been completely quantitatively synthesized and evaluated using knowledge outcomes in health education.

**Objective:**

The aim of this study was to assess the effectiveness of blended learning compared to that of traditional learning in health education.

**Methods:**

We performed a systematic review of blended learning in health education in MEDLINE from January 1990 to July 2019. We independently selected studies, extracted data, assessed risk of bias, and compared overall blended learning versus traditional learning, offline blended learning versus traditional learning, online blended learning versus traditional learning, digital blended learning versus traditional learning, computer-aided instruction blended learning versus traditional learning, and virtual patient blended learning versus traditional learning. All pooled analyses were based on random-effect models, and the I^2^ statistic was used to quantify heterogeneity across studies.

**Results:**

A total of 56 studies (N=9943 participants) assessing several types of learning support in blended learning met our inclusion criteria; 3 studies investigated offline support, 7 studies investigated digital support, 34 studies investigated online support, 8 studies investigated computer-assisted instruction support, and 5 studies used virtual patient support for blended learning. The pooled analysis comparing all blended learning to traditional learning showed significantly better knowledge outcomes for blended learning (standardized mean difference 1.07, 95% CI 0.85 to 1.28, I^2^=94.3%). Similar results were observed for online (standardized mean difference 0.73, 95% CI 0.60 to 0.86, I^2^=94.9%), computer-assisted instruction (standardized mean difference 1.13, 95% CI 0.47 to 1.79, I^2^=78.0%), and virtual patient (standardized mean difference 0.62, 95% CI 0.18 to 1.06, I^2^=78.4%) learning support, but results for offline learning support (standardized mean difference 0.08, 95% CI –0.63 to 0.79, I^2^=87.9%) and digital learning support (standardized mean difference 0.04, 95% CI –0.45 to 0.52, I^2^=93.4%) were not significant.

**Conclusions:**

From this review, blended learning demonstrated consistently better effects on knowledge outcomes when compared with traditional learning in health education. Further studies are needed to confirm these results and to explore the utility of different design variants of blended learning.

## Introduction

### Background

New types of learning, such as e-learning, have become popular in medical education [1,2] since the emergence of the internet [2]. These new models allow learning to transcend boundaries of space and time; they improve collaborative and individualized learning effectiveness and are more convenient [3-5]. Nevertheless, e-learning presents some disadvantages, including high cost multimedia materials, high costs for platform maintenance, and often user training is required. In parallel, traditional learning presents several limitations, including requiring the physical presence of students and teachers at a specific time and place [6].

Blended learning is characterized by the combination of traditional face-to-face learning and asynchronous or synchronous e-learning [7]. Blended learning is a promising alternative for medical education because of its advantages over traditional learning. In academia, this learning format has had a rapid growth and is now widely used [8].

Increased research on blended learning has been reported since the 1990s [9-11]. Synthesis of these studies may inform students and teachers on the effectiveness of blended learning [12]. Previous systematic reviews have reported that blended learning has the potential to improve clinical training among medical students [13] and undergraduate nursing education [14]. In parallel, many reviews have summarized the potential of blended learning in medical education [15,16]. A meta-analysis [12] showed that blended learning was more effective than nonblended learning but with a high level of heterogeneity.

Nevertheless, these reviews were limited to only some areas of health education, and few have used quantitative synthesis in the evaluation of the effectiveness of blended learning; therefore, the purpose of this study was to quantitatively synthesize the studies that evaluated the efficacy (using knowledge outcomes) of blended learning for health education (with students, postgraduate trainees, or practitioners).

### Objective

The objective of this review was to evaluate the effectiveness of blended learning for health education on knowledge outcomes assessed with subjective (eg, learner self-report) or objective evaluations (eg, multiple-choice question knowledge test) of learners’ factual or conceptual understanding of the course in studies where blended learning was compared with traditional learning.

## Methods

### Comparison Categories and Definitions

Blended learning was compared with traditional learning, overall, and after stratification by type of learning support, the following comparisons were made: offline blended learning versus traditional learning, online blended learning versus traditional learning, digital blended learning versus traditional learning, computer-aided instruction blended learning versus traditional learning, and virtual patient blended learning versus traditional learning.

Offline learning was defined as the use of personal computers or laptops to assist in delivering stand-alone multimedia materials without the need for internet or local area network connections [17]. These could be supplemented by videoconferences, emails, and audio-visual learning materials kept in either magnetic storage (CD-ROM, floppy disk, flash memory, multimedia cards, external hard disks) as long as the learning activities did not rely on this connection [18].

Online support was defined as all online materials used in learning courses.

Digital education was a broad construct describing a wide range of teaching and learning strategies that were exclusively based on the use of electronic media and devices as training, communication, and interactions tools [19]. These aspects could pertain to educational approaches, concepts, methods, or technologies. Moreover, these concepts facilitated remote learning, which could help address the shortage of health professionals in settings with limited resources by reducing the time constraints and geographic barriers to training.

Computer-assisted instruction was defined as the use of interactive CD-ROM, multimedia software, or audio-visual material to augment instruction including multimedia presentations, live synchronous virtual sessions offered via a web-based learning platform, presentations with audio-visuals, and synchronous or asynchronous discussion forums to enhance participation and increase engagement [20,21].

Virtual patients were defined as interactive computers simulations of real-life clinical scenarios for health professional training, education, or assessment. This broad definition encompassed a variety of systems that used different technologies and addressed various learning needs [22].

Traditional learning, in this paper, was used to describe all nonblended learning such as nondigital and not online, but also only online, only e-learning, or other single support educational methods (lectures, face-to-face, reading exercises, group discussion in classroom).

### Reporting Standards

We conducted and reported our study according to PRISMA guidelines [23] and Cochrane systematic review guidelines [24].

### Eligibility Criteria

Inclusion criteria for studies were based on the PICOS (population, intervention, comparison, outcome, and study design) framework.

Studies were included if they were conducted among health learners, used a blended learning intervention in the experimental group, involved a comparison of blended learning with traditional learning, included quantitative outcomes with respect to knowledge assessed with either subjective or objective evaluations, and were randomized controlled trials or nonrandomized studies (which are widely used in health education). Only studies published in English were included.

### Data Sources

To identify relevant studies, we conducted a search of citations published in MEDLINE between January 1990 and July 2019. Key search terms included delivery concepts (*blended, hybrid, integrated, computer-aided, computer assisted, virtual patient, learning, training, education, instruction, teaching, course*), participant characteristics (physician, medic*, nurs*, pharmac*, dent*, health*), and study design concepts (compar*, trial*, evaluat*, assess*, effect*, pretest*, pre-test, posttest*, post-test, preintervention, pre-intervention, postintervention, post-intervention). Asterisks were used as a truncation symbol for searching. Multimedia Appendix 1 describes the complete research strategy.

### Study Selection

Using the eligibility criteria, AV and ES independently screened all articles and abstracts and reviewed the full text of potentially eligible abstracts.

### Data Extraction

AV and ES independently extracted relevant characteristics related to participants, intervention, comparators, outcome measures, and results from the studies that were found to be eligible using a standard data collection form. Any disagreements were resolved through discussion with a third research team member until agreement was reached.

### Risk of Bias Assessment

During the data extraction process, researchers independently assessed the risk of bias for each study using the Cochrane Collaboration’s risk of bias tool [25]. Evaluation criteria included the following: random sequence generation, allocation concealment, blinding of students and personnel, blinding of outcome assessment, incomplete outcome data, selective reporting, or other which included publication bias. Funnel plots were used to evaluate publication bias. Risk of bias for each criterion was rate as low, high, or unclear according to the Cochrane risk of bias instructions.

### Data Synthesis

Analyses were performed for knowledge outcomes using SAS software (version 9.4; SAS Institute). The standardized mean difference (standard mean difference; Hedges g effect size), converted from means and standard deviation from each study, was used [15]. When the mean was available, but the standard deviation was not, we used the mean standard deviation of all other studies. Since the overall scores of included studies were not the same and standard mean difference could eliminate the effects of absolute values, we adjusted the mean and standard deviation so that the average standard deviation could replace the missing value of standard deviation. We employed a random-effects model for the meta-analysis (statistically significant if *P*<.05).

The I^2^ statistic was used to quantify heterogeneity across studies [26]. When the estimated I^2^ was equal to or greater than 50%, this indicated a large amount of heterogeneity. As the studies were functionally different and involved different study designs, participants, interventions, and settings; a random-effects model that allowed more heterogeneity was used. Forest plots were created to display the meta-analysis findings. To explore publication bias, funnel plots were created and Begg tests were performed (statistically significant if *P*<.05). To explore potential sources of heterogeneity, multiple meta-regression and subgroup analyses based on the study design were performed. Sensitivity analyses to test the robustness of findings were also performed.

## Results

### Study Selection

**Figure 1:**
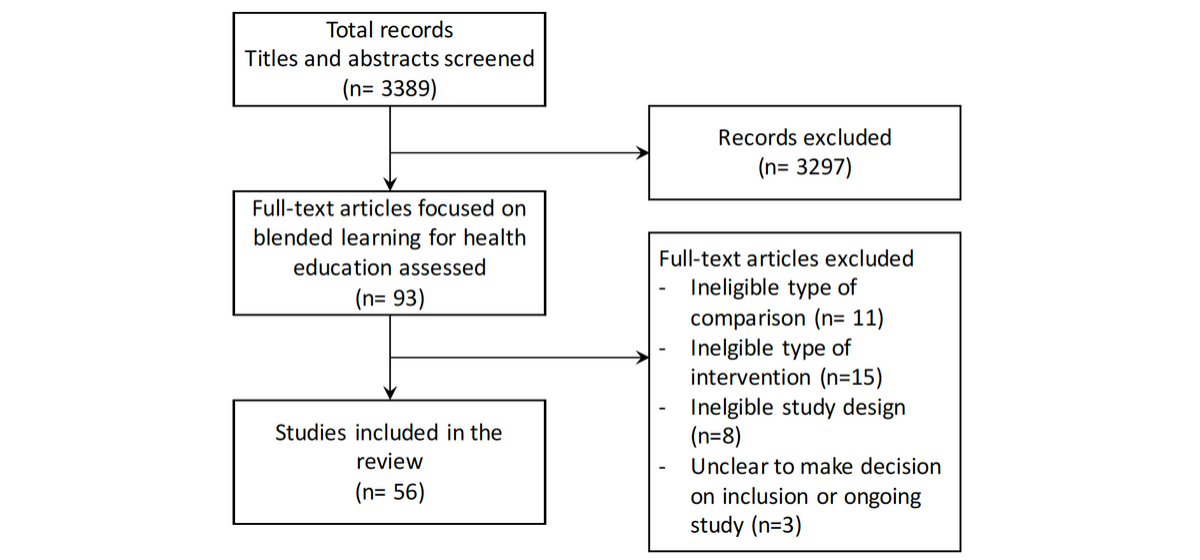
Study flow diagram.

The search strategy identified 3389 articles from MEDLINE. After scanning the titles and abstracts, 93 articles were found to be potentially eligible, and their full texts were read for further assessment. Of these, 56 articles were included [9-11,22,27-78] (Figure 1). All articles that were included had been published in peer-reviewed journal.

### Type of Participants

In the 56 articles, 9943 participants were included. In 30 out of 56 participant subgroups, participants were from the field of medicine [11,31-34,36,38,39,41,43,44,46-50,53,64-67,69-74, 76,78,79]. The participant subgroups from fields other than medicine were as follows: 16 studies in nursing [9,10,12,27,29,35,37,40,51,52,57,58,61-63,75], 1 in pharmacy [37], 3 in physiotherapy [12,30,45], 5 in dentistry [10,42, 54,55,59], and 4 interprofessional education [56,60,68,77].

Of the 56 studies, 47 were conducted in high-income countries: 14 were from the United States [9,10,29,31,36,38,43,50, 53-55,59,61,73], 2 from Canada [47,58], 5 from Germany [39,41,46,57,76], 3 from the United Kingdom [42,56,75], 3 from Spain [30,45,48], 1 from France [74], 1 from Greece [34], 1 from Sweden [67], 1 from the Netherlands [37], 1 from Korea [40], 1 from Poland [79], 1 from Serbia [70], 1 from Croatia [64], 1 from Turkey [32], 2 from Taiwan [28,51], 1 from Japan [69], and 7 from Australia [44,49,63,65,66,68,78]. Of the 56 studies, 9 studies were conducted in low- or middle-income countries: 2 from Thailand [52,62], 1 from China [77], 1 from Malaysia [72], 2 from Iran [27,71], 1 from Jordan [35], 1 from South Africa [11], and 1 from Uruguay [60].

The technical characteristics of the blended learning systems, topics of educational content, applied design methods, and other information on the validity of outcome measurements can be found in Multimedia Appendix 1.

### Effects of Interventions

#### Blended Learning Versus Traditional Learning

**Figure 2:**
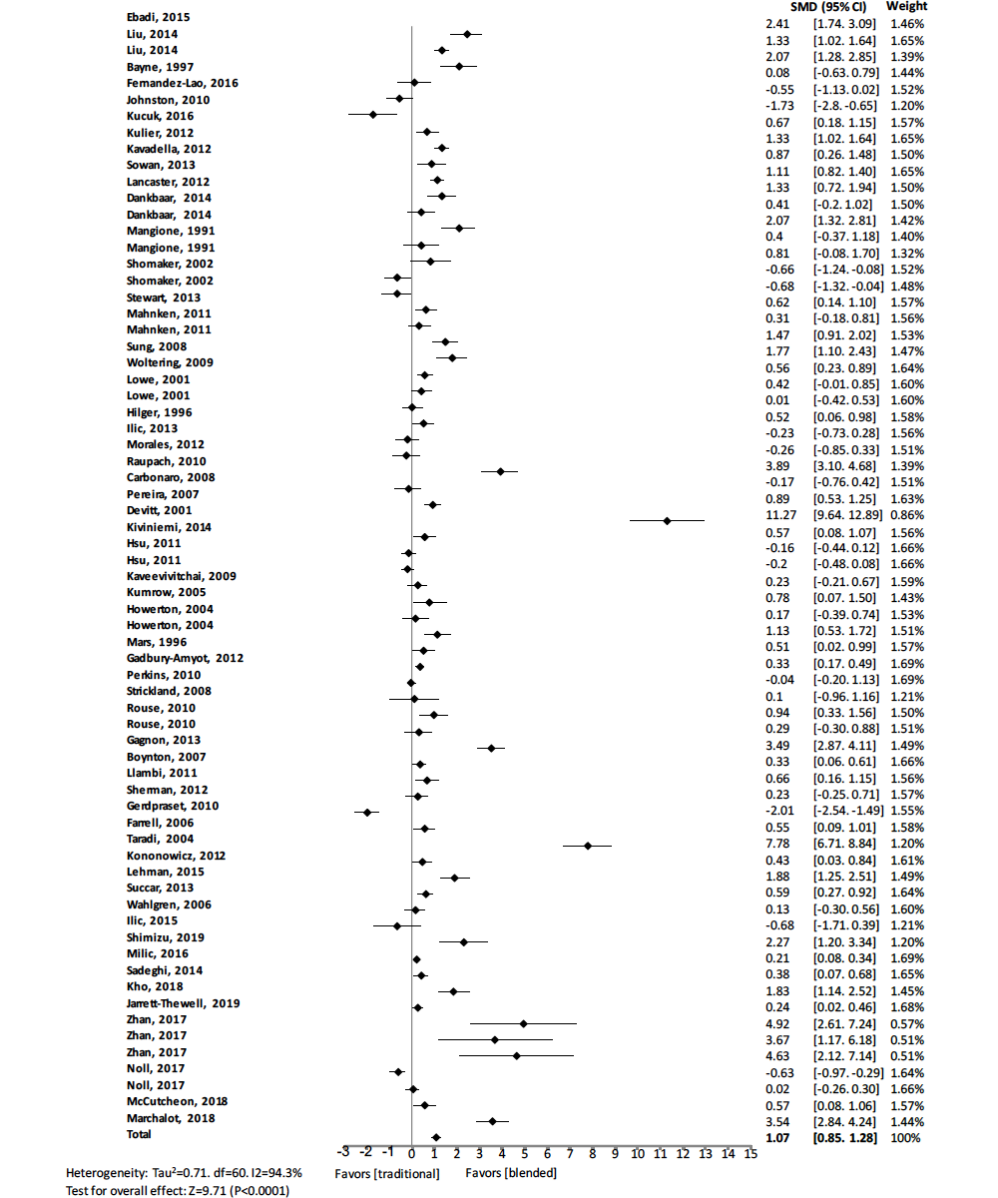
Forest plot of blended learning to traditional learning comparison for knowledge outcomes. df: degree of freedom; CI: confidence interval; SMD: standard mean difference.

**Figure 3:**
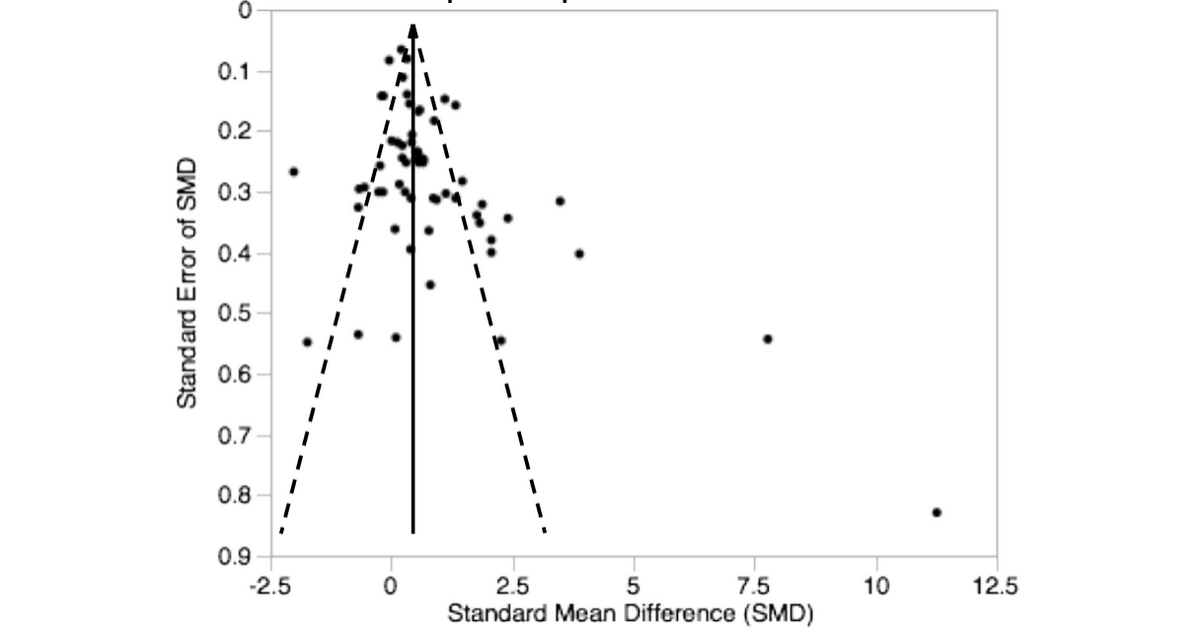
Funnel plot of blended learning versus traditional learning.

The pooled effect size reflected a significantly large effect on knowledge outcome (standard mean difference 1.07, 95% CI 0.85 to 1.28, *z=*9.72, n=9943, *P<*.001). A significant heterogeneity was observed among studies (I^2^=94.3%). Figure 2 shows details of the main analysis. The test of asymmetry funnel plot (Figure 3) indicated publication bias among studies (Begg test *P=*.01). The trim and fill method indicated that the effect size changed to 0.41 (95% CI 0.16 to 0.66, *P<*.001) after adjusting for publication bias, which suggested that blended learning was more effective than traditional learning.

#### Offline Blended Learning Versus Traditional Learning

**Figure 4:**
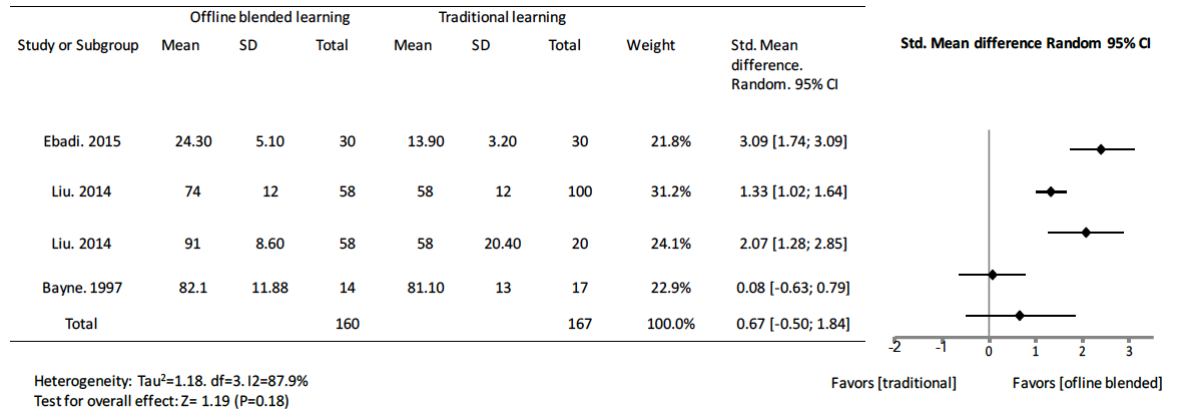
Forest plot of offline blended learning to traditional learning comparison for knowledge outcomes. df: degree of freedom; CI: confidence interval.

Of the 3 studies [27-29] comparing offline blended learning to traditional learning, in 2 studies [27,28], the groups with blended resources scored better than their corresponding control groups with significant positive standard mean differences. The other study did not show a statistically significant difference in knowledge outcome (standard mean difference 0.08, 95% CI –0.63 to 0.79) [29]. The pooled effect for knowledge outcomes suggested no significant effects from offline blended learning over traditional education alone (standard mean difference 0.67, 95% CI –0.50 to 1.84, I^2^=87.9%, n=327) (Figure 4).

#### Online Blended Learning Versus Traditional Learning

**Figure 5:**
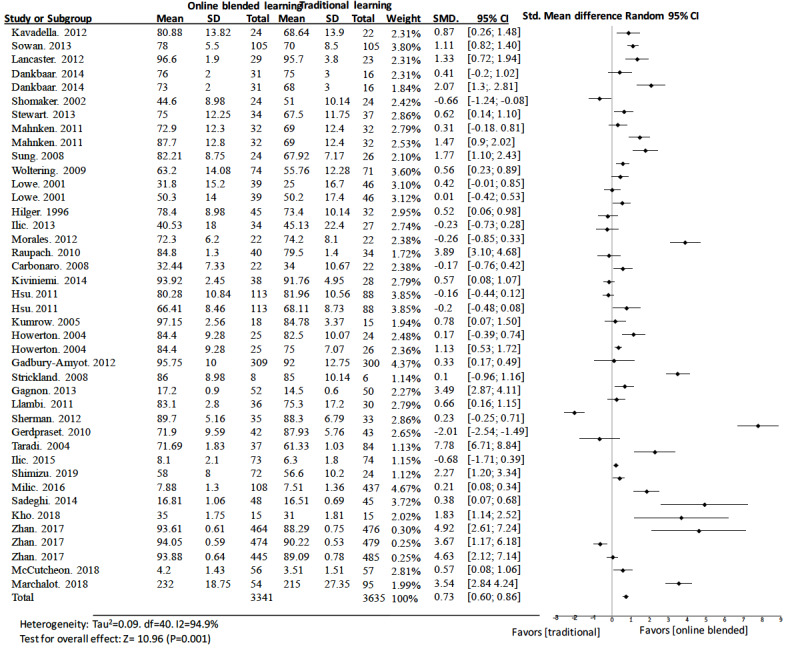
Forest plot of online blended learning to traditional learning comparison for knowledge outcomes. df: degree of freedom; CI: confidence interval; SMD: standard mean difference.

In studies comparing online blended learning to traditional learning, 26 [34-37,39-41,43,46,50,53-55,58,60,64,69-72, 74,75,77,78] of the 41 studies [34-47,50,51,53-55,57,58,60,62, 64,68-72,74,75,77,78] showed that groups with blended learning had better scores than those of their corresponding control groups. The pooled effect for knowledge outcomes was a standard mean difference of 0.73 (95% CI 0.60 to 0.86, n=6976) (Figure 5). There was a substantial amount of heterogeneity in the pooled analysis (I^2^=94.9%).

#### Digital Learning Versus Traditional Learning

**Figure 6:**
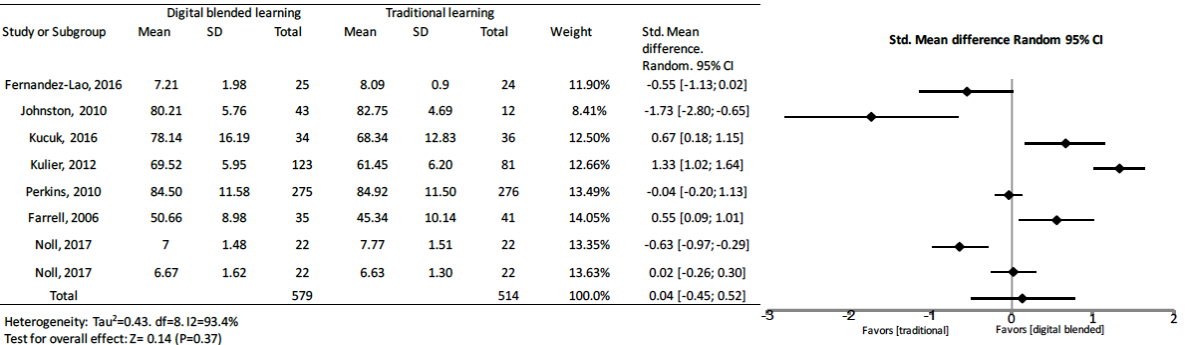
Forest plot of digital blended learning to traditional learning comparison for knowledge outcomes. df: degree of freedom; CI: confidence interval.

Only 3 [32,33,63] of 7 studies [30-33,56,63,76] comparing digital learning to traditional learning presented a better score than the control group. The pooled effect for knowledge outcomes suggested no significant effects between blended and traditional learning (standard mean difference 0.04, 95% CI –0.45 to 0.52, I^2^=93.4%, n=1093) (Figure 6).

#### Computer-Assisted Instruction Blended Learning Versus Traditional Learning

**Figure 7:**
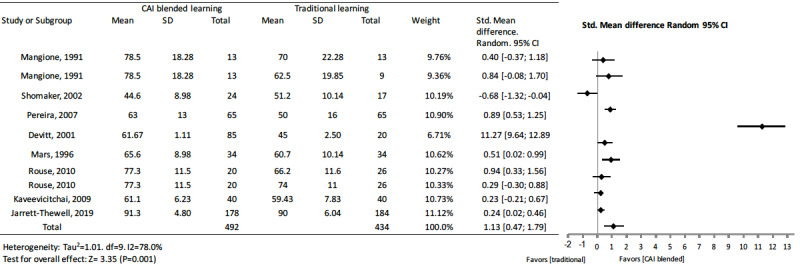
Forest plot of computer-assisted instruction blended learning to traditional learning comparison for knowledge outcomes. df: degree of freedom; CI: confidence interval.

Of the studies focusing on computer-assisted instruction blended learning, 5 [10,11,48,49,73] of the 8 studies [9-11,38,48, 49,52,73] showed significantly higher scores than those of traditional learning. Only 1 study [38] showed a significant negative effect compared to traditional learning (standard mean difference –0.68, 95% CI –1.32 to –0.04). The other studies showed no significant difference [9,52]. The pooled effect for knowledge outcomes suggested a significant improvement of computer-assisted instruction blended with traditional education over traditional education alone (standard mean difference 1.13, 95% CI 0.47 to 1.79, I^2^=78.0%, n=926) (Figure 7).

#### Virtual Patient Blended Learning Versus Traditional Learning

**Figure 8:**
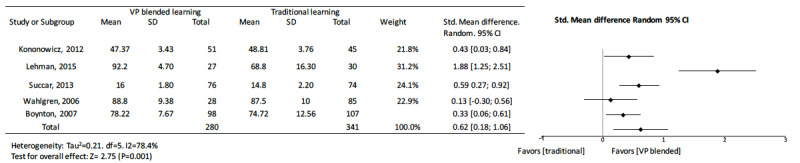
Forest plot of virtual patient blended learning to traditional learning comparison for knowledge outcomes. df: degree of freedom; CI: confidence interval.

In 4 [59,65,66,79] of the 5 studies [59,65-67,79] on knowledge outcomes when using virtual patients as a supplement to traditional learning, the groups with supplementary virtual patient learning support scored better than their corresponding control groups. Only 1 study with virtual patients did not show a statistically significant difference in knowledge outcomes (standard mean difference 0.13, 95% CI –0.30 to 0.56) [67]. The pooled effect for knowledge outcomes suggested significant effects for virtual patient blended learning (standard mean difference 0.62, 95% CI 0.18 to 1.06, I^2^=78.4%, n=621) (Figure 8).

### Sensitivity Analyses

None of the subgroup analyses that were initially planned explained the heterogeneity of the results. Among many analyzed aspects, we considered the differences regarding the efficiency of learning with blended learning between the health professions disciplines. Most of the studies involved students of medicine as participants (30/56, 54%).

**Figure 9:**
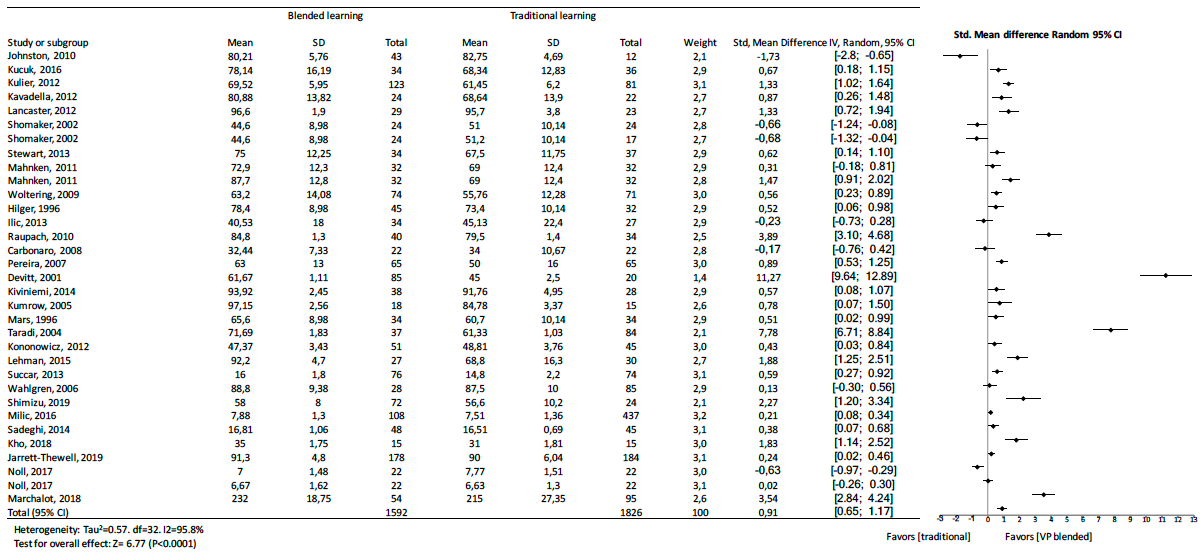
Forest plot of blended learning to traditional learning comparison for knowledge outcomes for medical students. df: degree of freedom; CI: confidence interval.

**Figure 10:**
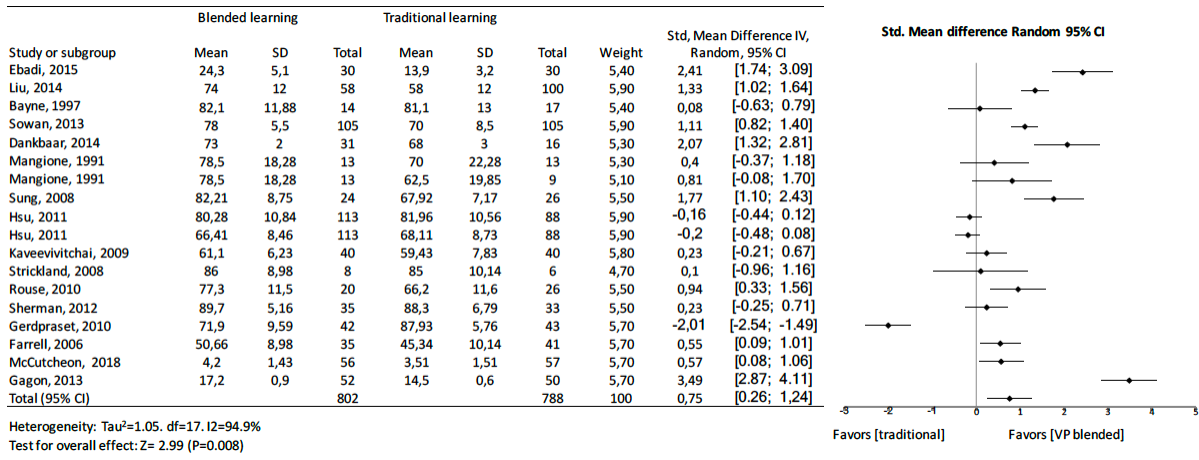
Forest plot of blended learning to traditional learning comparison for knowledge outcomes for nurses as students. df: degree of freedom; CI: confidence interval.

**Figure 11:**
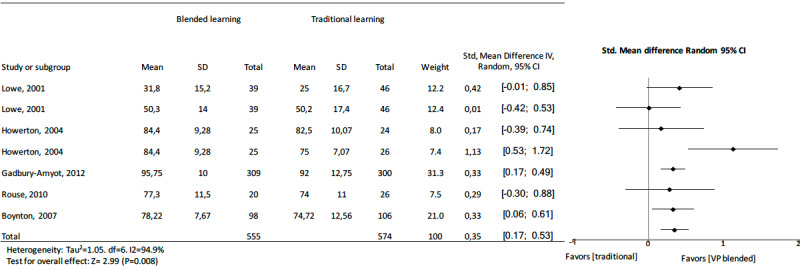
Forest plot of blended learning to traditional learning comparison for knowledge outcomes for dentistry students. df: degree of freedom; CI: confidence interval.

When analyzing knowledge outcomes in medicine, nursing, and dentistry, some differences were apparent. The pooled effect of medicine studies showed a standard mean difference of 0.91 (95% CI 0.65 to 1.17, *z=* 6.77, I^2^=95.8%, n=3418, *P<*.001) (Figure 9), nursing studies showed a standard mean difference of 0.75 (95% CI 0.26 to 1.24, *z=*2.99, I^2^=94.9%, n=1590, *P=*.008) (Figure 10), and dentistry studies showed a standard mean difference of 0.35 (95% CI 0.17 to 0.53, *z=*3.78, I^2^=37.6%, n=1130, *P=<*.001) (Figure 11). Dentistry studies included 3 online blended learning studies (standard mean difference 0.37, 95% CI 0.14 to 0.64, *z=*2.63, I^2^=58.3%, n=879), 1 virtual patient learning study, and 1 computer-assisted instruction learning study.

Additional interest was observed for offline blended learning in nursing compared to traditional learning (standard mean difference 1.28, 95% CI 0.25 to 2.31, *z=*2.43, I^2^=86.2%, n=249), and in computer-assisted instruction (standard mean difference 0.53, 95% CI 0.17 to 0.90, *z=*2.84, I^2^=23.9%, n=174), but not for online blended learning (standard mean difference 0.68, 95% CI –0.07 to 1.45, *z=*1.76, I^2^=96.7%, n=1091).

Additional interest was observed for digital blended learning compared to traditional learning in medicine (standard mean difference 0.26, 95% CI 0.07 to 0.45, *z=*2.71, I^2^=95.6%, n=417) [31-33,76], in virtual patient (standard mean difference 0.71, 95% CI 0.14 to 1.28, *z=*2.45, I^2^=85.8%, n=416) [65-67,79], in online (standard mean difference 1.26, 95% CI 0.81 to 1.71, *z=*5.49, I^2^=96.1%, n=1879) [34,36,38,39,41,43,44,46,47,50,53, 64,69-72,74,78], and in computer-aided-instruction (standard mean difference 2.1, 95% CI 0.68 to 3.44, *z=*2.91, I^2^=97.9%, n=706) [11,38,48,49,73] suggesting more positive effects of blended learning over traditional learning alone for learning in medicine.

### Risk of Bias

**Figure 12:**
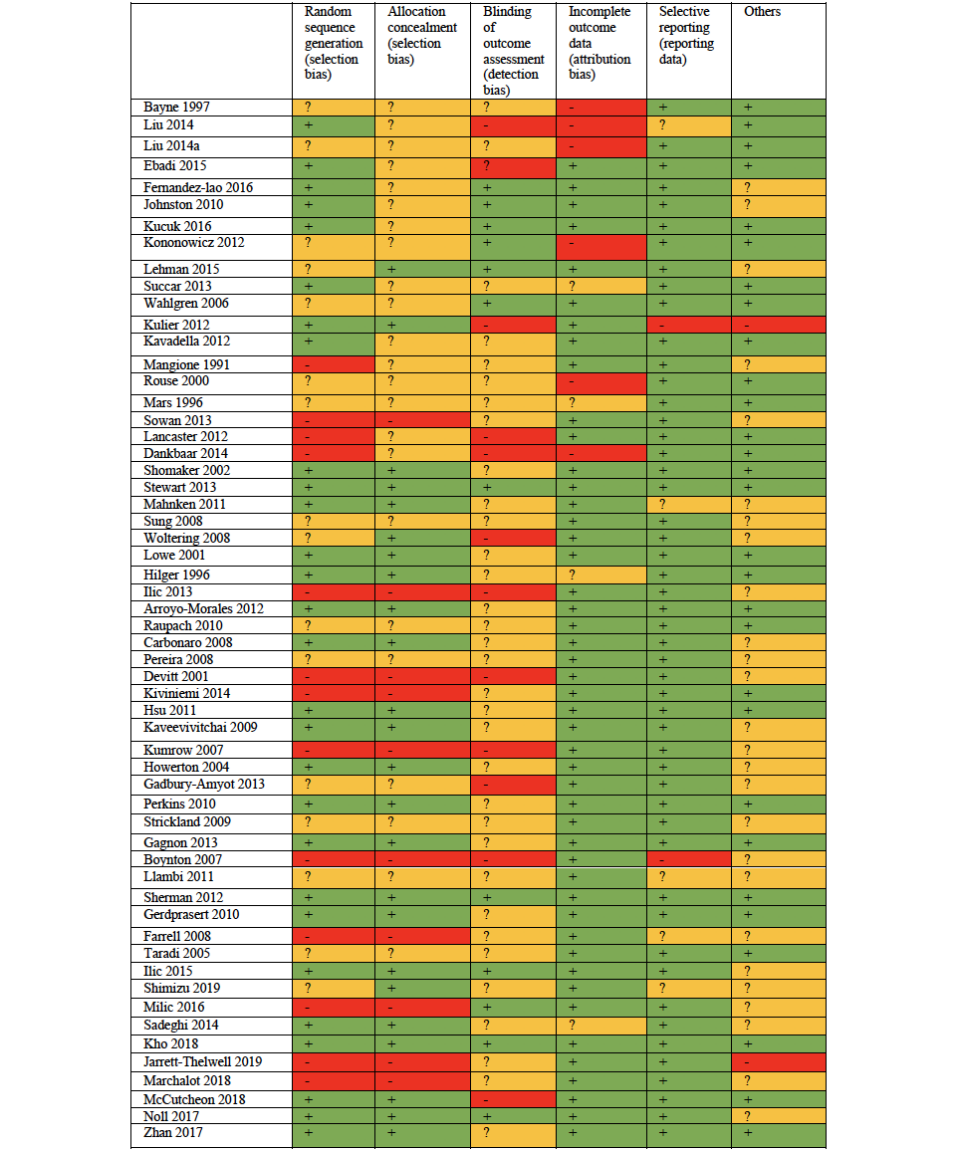
Risk of bias summary (+ low risk of bias; - high risk of bias; ? unclear risk of bias).

Risk of bias is shown in Figure 12. The risk of bias of evaluators was avoided in several studies by using automated assessment instruments. Thus, we rated the risk as low in 50 of the 56 studies. Nevertheless, it was still unclear whether the instruments had been correctly validated. Attribution bias was within acceptable levels in some studies (low risk in 24 of the 56 studies), but this did not exclude voluntary bias and its influence on the estimated effect. Reporting bias was considered low in 28 of the 56 studies.

We cannot consider allocation bias as a significant problem in this review because, if studies described an adequate randomization method or an unclear description, it was assumed that randomization was unlikely be defective. Performance bias on traditional learning may be a problem, but it is impossible to avoid in this type of research. It is possible to blind participants in blended learning design comparisons, but these studies are still rare in the literature. We cannot reliably estimate publication bias given the high degree of heterogeneity of the included studies.

## Discussion

### Principal Findings

This meta-analysis provided several findings. First, blended learning had a large consistent positive effect (standard mean difference 1.07, 95% CI 0.85 to 1.28) on knowledge acquisition in comparison to traditional learning in health professions. A possible explanation could be that, compared to traditional learning, blended learning allowed students to review electronic materials as often as necessary and at their own pace, and this likely enhanced learning performance [80]. The trim and fill method showed that the pooled effect size changed to 0.41 (95% CI 0.16 to 0.66), meaning that blended learning remained more effective than traditional learning. The strength of this meta-analysis was that it reinforced previous results [12]; however, a large heterogeneity was observed across the studies. The participant subgroup analyses partially explained these differences.

The effectiveness of blended learning is complex and dependent on how well the evaluation fits, since it occurs before the implementation of any innovation as well as allowing planners to determine the needs, considering participant characteristics, analyzing contextual matters, and gathering baseline information [81]. Some interventional studies have highlighted the potential of blended learning to increase course completion rates, improve retention, and increase student satisfaction [82]. Nevertheless, comparisons between blended learning environments and the disparity between academic achievement or grade dispersions have been studied; no significant differences were observed [83]. The effectiveness of blended learning may be dependent on student characteristics, design features, and learning outcomes. Learner success is dependent on the ability to cope with technical difficulty, technical skills, and knowledge in computer operations and internet navigation. Thus, the success of blended learning is highly dependent on experience with the internet and computer apps. Some studies have observed that the success of blended learning was largely associated on the capability to participate in blended course. A previous study [84] showed that high motivation among blended learning led to persistence in their courses. Moreover, time management is a crucial effectiveness factor for successful online learning [85].

Second, offline blended learning did not show a positive pooled effect compared to traditional learning; however, 2 of the 3 studies were in nursing. These results were consistent with a previous meta-analysis on offline digital education [86]. Nevertheless, potential benefits of offline education such as unrestrained knowledge transfer and enriched accessibility of health education have previously been suggested [87]. These interventions could be focused on an interactive, associative, and perceptual learning experience by text, images, audio-video, or other components [88,89].

Third, the effect of digital learning on knowledge outcomes presented inconsistent effects according to the group or subgroup analysis. Overall, the 8 digital blended learning studies showed a nonsignificant effect compared to traditional learning whereas in the medicine subgroup, digital learning had a positive effect (standard mean difference 0.26, 95% CI 0.07 to 0.45). Previous studies [18,90] have shown similar results. Nevertheless, George et al [18] showed the effectiveness of digital learning for undergraduate health professionals compared to traditional learning.

Fourth, in the 10 studies related to computer-assisted instruction, we observed a significant difference in knowledge acquisition outcomes. Furthermore, the difference was higher in the medicine subgroup. This finding must be interpreted with caution because of the high level of heterogeneity (all computer-assisted instruction: I^2^=78.0%; medicine computer-assisted instruction: I^2^=97.9%). Previous studies showed that computer-assisted instruction was equally as effective as traditional learning [91]. Nevertheless, the results of these studies also had high levels of heterogeneity and require cautious interpretation. We believe that a comparative approach focusing on the differences in intervention design, sample characteristics, and context of learning is needed to better understand the effectiveness of computer-assisted instruction. Computer-assisted instruction could be perceived negatively by some students and impact outcomes.

Fifth, the participants in Al-Riyami et al’s study [92] reported difficulties accessing the course because of network difficulties with university’s server and internet; therefore, the asynchronous features of the discussion boards were not used to their full potential in this study. Both problems could have emerged regardless of the online course. In traditional learning, students may choose not to engage in discussions, and internet connectivity issues can happen anywhere. This supports the contention above that local conditions, rather than a general effect, may render one or the other mode of instruction preferable to the other.

Sixth, virtual patient blended learning had an overall positive pooled effect when compared to traditional learning on knowledge outcomes; this was also found in a similar meta-analysis [93]. Our observations also supplement the evidence in previous reviews [94,95] which included studies since 2010. Nevertheless, virtual patient simulations predominantly affect skill rather than knowledge outcomes. This could explain the low number of studies and the low added value of virtual patient in comparison to traditional learning. Virtual patients have greater impact in skills training, in applying problem solving, and when direct patient contact is not possible [93]. As proposed by Cook and Triola [96], virtual patients can be said to be a modality for learning in which learners actively use and train their clinical reasoning and critical thinking abilities before bedside learning [96]. Nevertheless, some exceptions can be noted. A need for more guidance within virtual patient simulations may appear in studies with different instructional methods where narrative virtual patient design was better than more autonomous problem-oriented designs [97]. Feedback given by humans in a virtual patient system was better than an animated backstory in increasing empathy [98], but no feedback had no more positive result on the outcomes than learning from a virtual patient scenario [99]. This reminds us that presenting realistic patient scenarios with a great degree of freedom may not be an excuse for neglecting guidance in relation to learning objectives [100].

### Strengths and Limitations

This meta-analysis had numerous strengths. An evaluation of the effectiveness of blended learning for health professions is timely and very important for both health educators and learners. We intentionally kept our scope broad in terms of learning topic and included all studies with learners from health professions.

The samples used in this study consisted in various health professionals (in medicine, nursing, dentistry, and others) across a wide variety of health care disciplines. Although, these observations could explain the high level of heterogeneity, we found moderate or large effects for the pooled effects sizes of almost all subgroup analyses exploring variations in participant types. Thus, these results could suggest that health care learning should use blended learning in several and various disciplines of health learning.

However, some limitations must be considered. The systematic literature search encompassed one database (MEDLINE) with few exclusion criteria. The quality of the analyses was dependent on the quality of data from the included studies. Although the standard deviation of some interventions was not available due to poor reporting, we used the average standard deviation of the other studies and imputed effect sizes with concomitant potential for error. Results of subgroup analyses should be interpreted with caution because of the absence of a priori hypotheses in some cases, such as study design, country socioeconomic status, and outcome assessment. In addition, since variability of study interventions, assessment instruments, circumstances were not assessed and could be potential sources of heterogeneity, this should also be cause for cautious interpretation of results. Finally, publication bias was also found.

### Conclusions

This study has implications for research on blended learning in health professions. Even though conclusions could be weakened by heterogeneity across studies, the results of this synthesis reinforced that blended learning may have a positive effect on knowledge acquisition related to health professions. Blended learning could be promising and worthwhile for further application in health professions. The difference in effects across subgroup analyses of health population indicated that different methods of conducting blended courses could demonstrate differing effectiveness. Therefore, researchers and educators should pay attention to how to implement a blended course effectively. This question may be answered successfully through studies directly comparing different blended instructional methods.

## Appendix

Multimedia Appendix 1 Supplemental appendix.
