# Supplementary material for: Blended Learning Compared to Traditional Learning in Medical Education: Systematic Review and Meta-Analysis
Source: J Med Internet Res. 2020 Aug 10;22(8):e16504. doi: 10.2196/16504 (PMC7445617; doi:10.2196/16504)
Supplement: Multimedia Appendix 1 [file jmir_v22i8e16504_app1.docx]

## Multimedia Appendix 1. E-tables.

**E-table 1.search strategy**

***= wild word Adj=adjacent**

**Medline:**

1. “blended learning”[Title/Abstract] OR “hybrid learning”[Title/Abstract] OR “integrated learning”[Title/Abstract] OR “computer-aided learning”[Title/Abstract]

OR “computer-assisted learning”[Title/Abstract] OR “distributed learning”[Title/Abstract] OR “hybrid training”[Title/Abstract] OR “integrated

t raining”[Title/Abstract] OR “computer-aided t raining”[Title/Abstract] OR “integrated education”[Title/Abstract] OR “computer-aided education”[Title/Abstract] OR "computer-assisted education"[Title/Abstract] OR "distributed education"[Title/Abstract] OR “integrated instruction”[Title/Abstract] OR “computer-aided instruction”[Title/Abstract] OR "computer-assisted instruction"[Title/Abstract] OR “blended teaching”[Title/Abstract] OR “integrated teaching”[Title/Abstract]

OR “computer-aided teaching”[Title/Abstract] OR “computer-assisted teaching”[Title/Abstract] OR “blended course”[Title/Abstract] OR “hybrid course”[Title/Abstract] OR “integrated course”[Title/Abstract] OR“computer-assisted course”[Title/Abstract]

1. physician*[Title/Abstract] OR medic*[Title/Abstract] OR nurs*[Title/Abstract] OR pharmac*[Title/Abstract] OR dental[Title/Abstract] OR health*[Title/Abstract]
2. compar* OR trial* OR evaluat* OR assess* OR effect* OR pretest* OR pre-test OR posttest* OR post-test OR preinterven* OR pre-intervention OR postinterven* OR post-intervention

4.1 AND 2 AND 3

# E-Table 2. List of included publications

Arroyo-Morales, M., Cantarero-Villanueva, I., Fernández-Lao, C., Guirao-Piñeyro, M., Castro-Martín, E., and Díaz-Rodríguez, L. (2012). A blended learning approach to palpation and ultrasound imaging skills through supplementation of traditional classroom teaching with an e-learning package. *Man. Ther.* 17, 474–478. doi:10.1016/j.math.2012.04.002.

Bayne, T., and Bindler, R. (1997). Effectiveness of medication calculation enhancement methods with nurses. *J. Nurs. Staff Dev. JNSD* 13, 293–301.

Boynton, J. R., Green, T. G., Johnson, L. A., Nainar, S. M. H., and Straffon, L. H. (2007). The virtual child: evaluation of an internet-based pediatric behavior management simulation. *J. Dent. Educ.* 71, 1187–1193.

Carbonaro, M., King, S., Taylor, E., Satzinger, F., Snart, F., and Drummond, J. (2008). Integration of e-learning technologies in an interprofessional health science course. *Med. Teach.* 30, 25–33. doi:10.1080/01421590701753450.

Dankbaar, M. E. W., Storm, D. J., Teeuwen, I. C., and Schuit, S. C. E. (2014). A blended design in acute care training: similar learning results, less training costs compared with a traditional format. *Perspect. Med. Educ.* 3, 289–299. doi:10.1007/s40037-014-0109-0.

Devitt, P., Smith, J. R., and Palmer, E. (2001). Improved student learning in ophthalmology with computer-aided instruction. *Eye Lond. Engl.* 15, 635–639. doi:10.1038/eye.2001.199.

Ebadi, A., Yousefi, S., Khaghanizade, M., and Saeid, Y. (2015). Assessment Competency of Nurses in Biological Incidents. *Trauma Mon.* 20, e25607. doi:10.5812/traumamon.25607.

Farrell, M. J., and Rose, L. (2008). Use of mobile handheld computers in clinical nursing education. *J. Nurs. Educ.* 47, 13–19.

Fernández-Lao, C., Cantarero-Villanueva, I., Galiano-Castillo, N., Caro-Morán, E., Díaz-Rodríguez, L., and Arroyo-Morales, M. (2016). The effectiveness of a mobile application for the development of palpation and ultrasound imaging skills to supplement the traditional learning of physiotherapy students. *BMC Med. Educ.* 16, 274. doi:10.1186/s12909-016-0775-1.

Gadbury-Amyot, C. C., Singh, A. H., and Overman, P. R. (2013). Teaching with technology: learning outcomes for a combined dental and dental hygiene online hybrid oral histology course. *J. Dent. Educ.* 77, 732–743.

Gagnon, M.-P., Gagnon, J., Desmartis, M., and Njoya, M. (2013). The impact of blended teaching on knowledge, satisfaction, and self-directed learning in nursing undergraduates: a randomized, controlled trial. *Nurs. Educ. Perspect.* 34, 377–382.

Gerdprasert, S., Pruksacheva, T., Panijpan, B., and Ruenwongsa, P. (2010). Development of a web-based learning medium on mechanism of labour for nursing students. *Nurse Educ. Today* 30, 464–469. doi:10.1016/j.nedt.2009.10.007.

Hilger, A. E., Hamrick, H. J., and Denny, F. W. (1996). Computer instruction in learning concepts of streptococcal pharyngitis. *Arch. Pediatr. Adolesc. Med.* 150, 629–631.

Howerton, W. B., Enrique, P. R. T., Ludlow, J. B., and Tyndall, D. A. (2004). Interactive computer-assisted instruction vs. lecture format in dental education. *J. Dent. Hyg. JDH* 78, 10.

Hsu, L.-L., and Hsieh, S.-I. (2011). Effects of a blended learning module on self-reported learning performances in baccalaureate nursing students. *J. Adv. Nurs.* 67, 2435–2444. doi:10.1111/j.1365-2648.2011.05684.x.

Ilic, D., Hart, W., Fiddes, P., Misso, M., and Villanueva, E. (2013). Adopting a blended learning approach to teaching evidence based medicine: a mixed methods study. *BMC Med. Educ.* 13, 169. doi:10.1186/1472-6920-13-169.

Ilic, D., Nordin, R. B., Glasziou, P., Tilson, J. K., and Villanueva, E. (2015). A randomised controlled trial of a blended learning education intervention for teaching evidence-based medicine. *BMC Med. Educ.* 15, 39. doi:10.1186/s12909-015-0321-6.

Jarrett-Thelwell, F. D., Burke, J. R., Poirier, J.-N., and Petrocco-Napuli, K. (2019). A comparison of student performance and satisfaction between a traditional and integrative approach to teaching an introductory radiology course on the extremities. *J. Chiropr. Educ.* 33, 21–29. doi:10.7899/JCE-17-26.

Johnston, R., Hepworth, J., Goldsmith, M., and Lacasse, C. (2010). Use of iPod^TM^ technology in medical-surgical nursing courses: effect on grades. *Int. J. Nurs. Educ. Scholarsh.* 7, Article43. doi:10.2202/1548-923X.2092.

Kavadella, A., Tsiklakis, K., Vougiouklakis, G., and Lionarakis, A. (2012). Evaluation of a blended learning course for teaching oral radiology to undergraduate dental students. *Eur. J. Dent. Educ. Off. J. Assoc. Dent. Educ. Eur.* 16, e88-95. doi:10.1111/j.1600-0579.2011.00680.x.

Kaveevivitchai, C., Chuengkriankrai, B., Luecha, Y., Thanooruk, R., Panijpan, B., and Ruenwongsa, P. (2009). Enhancing nursing students’ skills in vital signs assessment by using multimedia computer-assisted learning with integrated content of anatomy and physiology. *Nurse Educ. Today* 29, 65–72. doi:10.1016/j.nedt.2008.06.010.

Kho, M. H. T., Chew, K. S., Azhar, M. N., Hamzah, M. L., Chuah, K. M., Bustam, A., et al. (2018). Implementing blended learning in emergency airway management training: a randomized controlled trial. *BMC Emerg. Med.* 18, 1. doi:10.1186/s12873-018-0152-y.

Kiviniemi, M. T. (2014). Effects of a blended learning approach on student outcomes in a graduate-level public health course. *BMC Med. Educ.* 14, 47. doi:10.1186/1472-6920-14-47.

Kononowicz, A. A., Krawczyk, P., Cebula, G., Dembkowska, M., Drab, E., Frączek, B., et al. (2012). Effects of introducing a voluntary virtual patient module to a basic life support with an automated external defibrillator course: a randomised trial. *BMC Med. Educ.* 12, 41. doi:10.1186/1472-6920-12-41.

Küçük, S., Kapakin, S., and Göktaş, Y. (2016). Learning anatomy via mobile augmented reality: Effects on achievement and cognitive load. *Anat. Sci. Educ.* 9, 411–421. doi:10.1002/ase.1603.

Kulier, R., Gülmezoglu, A. M., Zamora, J., Plana, M. N., Carroli, G., Cecatti, J. G., et al. (2012). Effectiveness of a clinically integrated e-learning course in evidence-based medicine for reproductive health training: a randomized trial. *JAMA* 308, 2218–2225. doi:10.1001/jama.2012.33640.

Kumrow, D. E. (2007). Evidence-based strategies of graduate students to achieve success in a hybrid Web-based course. *J. Nurs. Educ.* 46, 140–145.

Lancaster, J. W., Wong, A., and Roberts, S. J. (2012). “Tech” versus “talk”: a comparison study of two different lecture styles within a Master of Science nurse practitioner course. *Nurse Educ. Today* 32, e14-18. doi:10.1016/j.nedt.2011.09.018.

Lehmann, R., Thiessen, C., Frick, B., Bosse, H. M., Nikendei, C., Hoffmann, G. F., et al. (2015). Improving Pediatric Basic Life Support Performance Through Blended Learning With Web-Based Virtual Patients: Randomized Controlled Trial. *J. Med. Internet Res.* 17, e162. doi:10.2196/jmir.4141.

Liu, W.-I., Chu, K.-C., and Chen, S.-C. (2014). The development and preliminary effectiveness of a nursing case management e-learning program. *Comput. Inform. Nurs. CIN* 32, 343–352. doi:10.1097/CIN.0000000000000050.

Llambí, L., Esteves, E., Martinez, E., Forster, T., García, S., Miranda, N., et al. (2011). Teaching tobacco cessation skills to Uruguayan physicians using information and communication technologies. *J. Contin. Educ. Health Prof.* 31, 43–48. doi:10.1002/chp.20100.

Lowe, C. I., Wright, J. L., and Bearn, D. R. (2001). Computer-aided Learning (CAL): an effective way to teach the Index of Orthodontic Treatment Need (IOTN)? *J. Orthod.* 28, 307–311. doi:10.1093/ortho/28.4.307.

Mahnken, A. H., Baumann, M., Meister, M., Schmitt, V., and Fischer, M. R. (2011). Blended learning in radiology: is self-determined learning really more effective? *Eur. J. Radiol.* 78, 384–387. doi:10.1016/j.ejrad.2010.12.059.

Mangione, S., Nieman, L. Z., Greenspon, L. W., and Margulies, H. (1991). A comparison of computer-assisted instruction and small-group teaching of cardiac auscultation to medical students. *Med. Educ.* 25, 389–395.

Marchalot, A., Dureuil, B., Veber, B., Fellahi, J.-L., Hanouz, J.-L., Dupont, H., et al. (2018). Effectiveness of a blended learning course and flipped classroom in first year anaesthesia training. *Anaesth. Crit. Care Pain Med.* 37, 411–415. doi:10.1016/j.accpm.2017.10.008.

Mars, M., and McLean, M. (1996). Students’ perceptions of a multimedia computer-aided instruction resource in histology. *South Afr. Med. J. Suid-Afr. Tydskr. Vir Geneeskd.* 86, 1098–1102.

McCutcheon, K., O’Halloran, P., and Lohan, M. (2018). Online learning versus blended learning of clinical supervisee skills with pre-registration nursing students: A randomised controlled trial. *Int. J. Nurs. Stud.* 82, 30–39. doi:10.1016/j.ijnurstu.2018.02.005.

Milic, N. M., Trajkovic, G. Z., Bukumiric, Z. M., Cirkovic, A., Nikolic, I. M., Milin, J. S., et al. (2016). Improving Education in Medical Statistics: Implementing a Blended Learning Model in the Existing Curriculum. *PloS One* 11, e0148882. doi:10.1371/journal.pone.0148882.

Noll, C., von Jan, U., Raap, U., and Albrecht, U.-V. (2017). Mobile Augmented Reality as a Feature for Self-Oriented, Blended Learning in Medicine: Randomized Controlled Trial. *JMIR MHealth UHealth* 5, e139. doi:10.2196/mhealth.7943.

Pereira, J., Palacios, M., Collin, T., Wedel, R., Galloway, L., Murray, A., et al. (2008). The impact of a hybrid online and classroom-based course on palliative care competencies of family medicine residents. *Palliat. Med.* 22, 929–937. doi:10.1177/0269216308094561.

Perkins, G. D., Fullerton, J. N., Davis-Gomez, N., Davies, R. P., Baldock, C., Stevens, H., et al. (2010). The effect of pre-course e-learning prior to advanced life support training: a randomised controlled trial. *Resuscitation* 81, 877–881. doi:10.1016/j.resuscitation.2010.03.019.

Raupach, T., Münscher, C., Pukrop, T., Anders, S., and Harendza, S. (2010). Significant increase in factual knowledge with web-assisted problem-based learning as part of an undergraduate cardio-respiratory curriculum. *Adv. Health Sci. Educ. Theory Pract.* 15, 349–356. doi:10.1007/s10459-009-9201-3.

Rouse, D. P. (2000). The effectiveness of computer-assisted instruction in teaching nursing students about congenital heart disease. *Comput. Nurs.* 18, 282–287.

Sadeghi, R., Sedaghat, M. M., and Sha Ahmadi, F. (2014). Comparison of the effect of lecture and blended teaching methods on students’ learning and satisfaction. *J. Adv. Med. Educ. Prof.* 2, 146–150.

Sherman, H., Comer, L., Putnam, L., and Freeman, H. (2012). Blended versus lecture learning: outcomes for staff development. *J. Nurses Staff Dev. JNSD Off. J. Natl. Nurs. Staff Dev. Organ.* 28, 186–190. doi:10.1097/NND.0b013e31825dfb71.

Shimizu, I., Nakazawa, H., Sato, Y., Wolfhagen, I. H. A. P., and Könings, K. D. (2019). Does blended problem-based learning make Asian medical students active learners?: a prospective comparative study. *BMC Med. Educ.* 19, 147. doi:10.1186/s12909-019-1575-1.

Shomaker, T. S., Ricks, D. J., and Hale, D. C. (2002). A prospective, randomized controlled study of computer-assisted learning in parasitology. *Acad. Med. J. Assoc. Am. Med. Coll.* 77, 446–449.

Sowan, A. K., and Jenkins, L. S. (2013). Use of the seven principles of effective teaching to design and deliver an interactive hybrid nursing research course. *Nurs. Educ. Perspect.* 34, 315–322.

Stewart, A., Inglis, G., Jardine, L., Koorts, P., and Davies, M. W. (2013). A randomised controlled trial of blended learning to improve the newborn examination skills of medical students. *Arch. Dis. Child. Fetal Neonatal Ed.* 98, F141-144. doi:10.1136/archdischild-2011-301252.

Strickland, S. (2009). The effectiveness of blended learning environments for the delivery of respiratory care education. *J. Allied Health* 38, E11-16.

Succar, T., Zebington, G., Billson, F., Byth, K., Barrie, S., McCluskey, P., et al. (2013). The impact of the Virtual Ophthalmology Clinic on medical students’ learning: a randomised controlled trial. *Eye Lond. Engl.* 27, 1151–1157. doi:10.1038/eye.2013.143.

Sung, Y. H., Kwon, I. G., and Ryu, E. (2008). Blended learning on medication administration for new nurses: integration of e-learning and face-to-face instruction in the classroom. *Nurse Educ. Today* 28, 943–952. doi:10.1016/j.nedt.2008.05.007.

Taradi, S. K., Taradi, M., Radic, K., and Pokrajac, N. (2005). Blending problem-based learning with Web technology positively impacts student learning outcomes in acid-base physiology. *Adv. Physiol. Educ.* 29, 35–39. doi:10.1152/advan.00026.2004.

Wahlgren, C.-F., Edelbring, S., Fors, U., Hindbeck, H., and Ståhle, M. (2006). Evaluation of an interactive case simulation system in dermatology and venereology for medical students. *BMC Med. Educ.* 6, 40. doi:10.1186/1472-6920-6-40.

Woltering, V., Herrler, A., Spitzer, K., and Spreckelsen, C. (2009). Blended learning positively affects students’ satisfaction and the role of the tutor in the problem-based learning process: results of a mixed-method evaluation. *Adv. Health Sci. Educ. Theory Pract.* 14, 725–738. doi:10.1007/s10459-009-9154-6.

Zhan, X., Zhang, Z., Sun, F., Liu, Q., Peng, W., Zhang, H., et al. (2017). Effects of Improving Primary Health Care Workers’ Knowledge About Public Health Services in Rural China: A Comparative Study of Blended Learning and Pure E-Learning. *J. Med. Internet Res.* 19, e116. doi:10.2196/jmir.6453.

# E-Table 3. Description of included publications

**Section 1. Studies comparing blended learning with traditional learning**

| **Study** | **Design RCT/NRS)** | **Country** | **Comparison intervention** | **Participants no.(B/Na); type** | **Topic** | **Study intervention(component or features)** | **Modality or technology** | **Duration** | **Exercises** | **Interactivity** | **Discussion** | **courseDelay between posttest and** | **Assessment(question type)** | **Conflict of interest;** | **Source of support** |
| --- | --- | --- | --- | --- | --- | --- | --- | --- | --- | --- | --- | --- | --- | --- | --- |
| Kulier, 2012 | Pre- posttes t 2 groups  ;RCT | 7  LMICs  (Argen t ina,  Brazil, Democ ratic Repub l ic of  the | Tradition al teaching | 123/81;  Postgraduat e t rainees | Reproductiv e Health | E-  learning+F2F  ODE | recorded video, specialist database | 8  week s | Present(questio ns, assignments) | High(Feedb ack on assignments  ) | Abs ent | 4  we eks | Objective(MCQ) | No | No |

|  |  | Congo, India, Philip pines, South Africa, Thaila nd). |  |  |  |  |  |  |  |  |  |  |  |  |  |
| --- | --- | --- | --- | --- | --- | --- | --- | --- | --- | --- | --- | --- | --- | --- | --- |
| Kavadella,2012 | Pre- posttes t 2 group; RCTs | Greece | Conventional face to face methodology | 24/22;  Undergradu ate | Oral radiology | F2F + online | E-learning platform Web  -based tools include self- graded tests and quizzes, online discussion  groups | 0.5  year | Present(self- graded tests and quizzes) | High(self- graded tests and quizzes, online discussion groups) | Pre sent | No del ay | Objective(dichoto mal :yes/no) | No | No |
| Sowan,2013 | Posttes t, 2 groups  ; RCT | Jordan | Tradition al format | 105/105;  undergradu ate nursing students | Scientific research in nursing | Web- based+interacti  ve F2F | Blackboard and Tegrity systems | 1  seme ster | Present(questio ns, assignments) | High (questions, assignments  ) | Pre sent | No del ay | Objective(open- ended questions) | No | No |
| Lancaster,2012 | Posttes t, 2 groups  ;RCT | USA | Tradition al in-class | 29/23;  Graduate | Pharmacoth erapeutics course | Oline+F2F | Griffin Lapel Microphone, Articulate Presenter '09, electronic  Blackboardhos t ing website | 1  year | Present(assign ment, question and answer sessions) | High (assignment  , question and answer session  ,question and answer  sessions) | Pre sent | No del ay | Objective(Cannot tell) | No | No |
| Dankbaar,2014(a  ) | Posttes t, 2 groups  ;NRS | Nether lands | Tradition al course | 31/16; Nurse in postgraduat e | Acute and intensive care | Online material+F2F  lecture | Web lectures | 11  days | Present (examples and exercises) | Low(exampl es and exercises  with  feedback) | Abs ent | No del ay | Objective(MCQ) | No | No |

| Dankbaar,2014(c  ) | Posttes t, 2 groups  ;NRS | Nether lands | Tradition al course | 31/16; Nurse in postgraduat e | Acute and intensive care | Online material+F2F  lecture | Web lectures | 11  days | Present (examples and exercises) | Low(exampl es and exercises  with  feedback) | Abs ent | No del ay | Objective(MCQ) | No | No |
| --- | --- | --- | --- | --- | --- | --- | --- | --- | --- | --- | --- | --- | --- | --- | --- |
| Mangione,1991(a  ) | Pre- posttes t, 2 groups  ;RCT | USA | Computer- assisted instructio n | 13/9;  Medical students | Cardiac auscultation | Self-schedule CAI + small- group seminar | HEARTLAB  platform, | 12  week s | Absent | Low | Abs ent | No del ay | Objective(choice question) | No | No |
| Mangione,1991(b  ) | Pre- posttes t, 2 groups  ;RCT | USA | Tutorial instructio n | 13/13;  Medical students | Cardiac auscultation | Self-schedule CAI + small- group seminar | HEARTLAB  platform, | 12  week s | Absent | Low | Abs ent | No del ay | Objective(choice question) | No | No |
| Shomaker, 2002(a) | Pre- osttest, 2  groups  ; RCT | *USA* | t raditiona l | 24/24;medic al students | parasitology | computer program + lectures | interactive text | 2  week s | Present(questio ns) | Low(questio ns) | Abs ent | No del ay | Objective(MCQ or slides) | No | No |
| Shomaker, 2002(b) | Pre- posttes t, 2 groups  ; RCT | *USA* | e-learning | 24/17;  medical students | parasitology |  | interactive text | 2  week s | Present(questio ns) | Low(questio ns) | Abs ent | No del ay | Objective(MCQ or slides) | No | No |
| Stewart,2013 | Posttes t, 2 groups  ; RCT | Austra l ia | standard teaching | 34/37;  Medical students | Newborn | Online module+standa rd programme | PENSKE Baby Check Learning module | 8  week s | Absent | Low | Abs ent | No del ay | Objective(Cannot tell) | No | No |
| Mahnken,2011(a  ) | Pre- posttes t, 2 groups  ; RCT | Germa ny | Tradition al  learning | 32/32;  Medical students | Radiology | E-  learning+intern ship(F2F) | Electronic cases | 1  week | Present (cases and expert feedback, question-and- answer | High (cases and expert feedback, question- and-answer) | Abs ent | No del ay | Objective(Cannot tell) | No | No |
| Mahnken,2011(b  ) | Pre- posttes t, 2 groups  ; RCT | Germa ny | Tradition al  learning | 32/32;  Medical students | Radiology | E-  learning+intern ship(F2F) | Electronic cases | 1  week | Present (cases and expert feedback, question-and- answer | High (cases and expert feedback, question- and-answer) | Abs ent | No del ay | Objective(Cannot tell) | No | No |
| Sung, 2008 | Pre- posttes t, 2 groups  ;NRS | Korea | Face to face  instructio n | 24/26;  Nurses | Medical administrati on | Web-based matirilas  +_face-to-face instruction | Web-based e- learning program | 10  mont hs | Present(quizzes with feedback, clinical cases) | High (quizzes with feedback, clinical  cases, active interaction between  tutors and  students) | Abs ent | No del ay | Objective(Cannot tell) | No | No |
| Woltering,2009 | Posttes | Germa | t raditiona | 74/71; | Model | Online | multimedia | 2 | Present(questio | High(online | Pre | No | Objective(MCQ) | No | No |

|  | t, 2 groups  ;NRS | ny | l PBL | Medical students | Curriculum Medicine | learning+stude nts’  Meeting+tutore d final session | case vignette, Group-Wiki, The virtual clinical order entry system, bulletin board | week s | ns, cases) | collaboratio n including comments of the tutor) | sent | del ay |  |  |  |
| --- | --- | --- | --- | --- | --- | --- | --- | --- | --- | --- | --- | --- | --- | --- | --- |
| Lowe,2001(a) | Posttes t, 2 groups  ;NRS | UK | lecture and seminar | 39/46;  Undergradu ate dental students | Index of Orthodontic t reatment  need | CAL  programme+se minar | Internet web- authoring package | 1  week | Present(self- assessment) | High(multi media design with interactive comment) | Pre sent | No del ay | Objective(cases) | No | No |
| Lowe,2001(b) | Posttes t, 2 groups  ;NRS | UK | lecture and seminar | 39/46;  Undergradu ate dental students | Index of Orthodontic t reatment  need | CAL  programme+se minar | Internet web- authoring package | 1  week | Present (self- assessment) | High(multi media design with interactive comment) | Pre sent | No del ay | Objective(cases) | No | No |
| **Hilger, 1996** | Pre- posttes t, 2 groups  ; RCT | *USA* | t raditiona l | 45/32;medic al students | Streptococca l  Pharyngitis | CAI program  +clerkship | Online  tutorial, case simulation | 4  week s | Present (case simulation,self- assessment) | High(discus sion with feedback) | Pre sent | No del ay | Objective(MCQ. True or false) | No | No |
| I l ic,2013 | Posttes t, 2 groups  ;NRS | Austra l ia | Didactic learning | 34/27;  Graduate medical students | Evidence based practice (EBP) | Tutorial sessions+web- site learning | Monash University  l ibrary website | 1  day | Present (patient-  basedPresentat ion) | High(group work,  patient- based Presentatio  n) | Pre sent | No del ay | Objective(MCQ) | Ye s | No |
| Morales,2012 | Posttes t, 2 groups, RCT | Spain | document s and books | 22/22;  Physiothera py second-year degree students | Physiothera py degree course | on-campus t raining+ website | ECOFISIO  website | 1  seme ster | Present(self- assessment) | Low(self- assessment) | Abs ent | No  del ay | Objective(MCQ) | No | No |

| Raupach,2010 | Pre- posttes t, 2 groups  ; RCT | Germa ny | Tradition al  learning | 40/34;  Medical students | Cardio- respiratory curriculum | Online module+traditi onal curriculum | web-based learning management system | 6  week s | Present(test with feedback) | High(test with feedback) | Pre sent | No del ay | Objective(MCQ) | No | No |
| --- | --- | --- | --- | --- | --- | --- | --- | --- | --- | --- | --- | --- | --- | --- | --- |
| Carbonaro,2008 | Pre- posttes t, 2 groups  ; RCT | Canad a | face to face= | 22/22;Stude nt | Health science program | E-  learning+F2F interprofession al team course |  | 5  week s | Present(giving/ receiving feedback, consensus decision- making) | High(giving/ receiving feedback, consensus decision- making, Group discussions, problem solving) | Pre sent | No del ay | Subjective(canno t tell) | No | No |
| Pereira,2007 | Posttes t, 2 groups  ;NRS | Spain | Tradition al teaching | 65/65;Stude nts | Human anatomy | Online learning+semin  ars | Computerized materials | 45  class hour s | Present(interac t ive multiple- choice, short- answer self- assessment test problem solving  activities) | High(intera ctive  multiple- choice, short-  answer sel f- assessment  test | Pre sent | No del ay | Objective(MCQ, short answer question, practical  question) | No | No |
| Devitt,2001 | Pre- posttes t, 2 group; nrss | Austra l ia | Lecture | 85/20;  Medical students | Ophthalmology ogy | Lecture+e- learning | Medici software | 2  week s | Present(cases) | Low | Abs ent | No del ay | Objective(MCQ) | No | No |
| Kiviniemi,2014 | Posttes t 2- group; NRS | USA | t raditiona l learning | 38/28;  Public health graduate student | Public health | Online lecture presentation  +didactic lecture | web | 3  week s | Absent | High(active learning activity) | Pre sent | No del ay | Objective(MCQ, short answer question) | No | No |
| Hsu,2011(a) | Pre- posttes t, 2 groups  ;NRS | Taiwa n | traditiona l learning | 113/88;  Nursing students | Nursing ethics | web-based teaching/learni ng module+ classroom lectures | web-based module (videos,  PowerPoint fi les) | 17  week s | Present (questio ns and comments) | High(excha nge ideas, questions and comments) | Pre sent | No del ay | Objective(cannot tell) | No | No |
| Hsu,2011(b) | Pre- posttes t, 2 group; | Taiwa n | traditiona l learning | 113/88;  Nursing students | Nursing ethics | web-based teaching/learning module+ classroom | web-based module (videos,  PowerPoint  fi les) | 17  week s | Present(questio ns and comments) | High(excha nge ideas, questions and | Pre sent | No del ay | Subjective(canno t tell) | No | No |

| Kaveevivitchai,2 009 | Pre- posttes t, 2 group; RCT s | Thaila nd | t raditiona l learning | 40/40;Nursi ng students | Anatomy and physiology | Lectures CAL  multimedia+tra ditional lecture | interactive CAL  multimedia | 2  days | Present(questio ns, case scenarios) | High(questi ons, case scenarios) | Pre sent | No del ay | Objective(MCQ) | No | No |
| --- | --- | --- | --- | --- | --- | --- | --- | --- | --- | --- | --- | --- | --- | --- | --- |
| Kumrow,2005 | Posttes t 2- group; NRS | USA | t raditiona l learning | 18/15;  Graduate nursing students | Health care economic policy and managemen t | Online  instruction(50%  )+traditional in- classface-to- face(50%) | Web-based | >1  seme ster | Absent | Low | Abs ent | No del ay | Subjective(self- report) | No | No |
| Howerton,2004(a  ) | Pre- posttes t, 2 groups  ; RCT | USA | t raditiona l learning | 25/24;  Dental students | Dental radiology | Interactive CD  +lecture | Director 8 authoring software | 2  week s | Present(exercis es) | High(exercis es,  interactive presentatio  n) | Abs ent | 2  we eks | Objective(cannot tell) | No | No |
| Howerton,2004(b  ) | Pre- posttes t, 2 groups  ; RCT | USA | e-learning | 25/26;  Dental students | Dental radiology | Interactive CD  +lecture | Director 8 authoring software | 2  week s | Present(exercis es) | High(exercis es,  interactive presentatio  n) | Abs ent | 2  we eks | Objective(cannot tell) | No | No |
| Mars,1996 | Pre- posttes t, 2 groups  ;NRS | Durba n | traditional learning | 34/34;  Medical students | histology | CAI module+ | onscreen “patient” | 3  week s | Present(self- assessment questions | High (self- assessment questions  ,asking and answering  questions) | Abs ent | No del ay | Objective(cannot tell) | No | No |
| Gadbury- Amyot,2012 | Posttes t 2- group; NRS | USA | traditiona l learning | 309/300;  Dental and dental hygiene students | Oral  Histology | CAI+lecture | Software sta ndard  interactions | >1  seme ster | Present(questio ns, self- assessment) | High(intera ctive  multimedia) | Abs ent | No del ay | Objective(cannot tell) | No | No |
| Perkins,2010 | Pre- posttes t, 2 groups  ; RCT | UK | traditiona l learning | 275/276;  Medical students | Life support | Face-to-face course +e- learning  ODE | Microsim programme on a CD | 4  week s | Absent | Low(Feedba ck on  experiences) | Abs ent | No del ay | Objective(MCQ) | Ye s | No |
| Strickland,2008[ 6][6][26][6] | Pre- posttes t, 2 groups | Germa n | traditiona l learning | 8/6; Health professions student | Respiratory Care | Course materials via  Internet +face-  to-face | Cannot tell | 1  seme ster | Absent | Low | Abs ent | No del ay | Objective(cannot tell) | No | No |
| Rouse,2000(a) | Pre- posttes t, 2 groups  ; RCT | USA | traditiona l learning | 20/26;  Nursing Students | Pediatric nursing | computer- assisted  instruction  +traditional class room lecture | CD-ROM,  computer | >1  seme ster | Absent | Low | Abs ent | No del ay | Objective(MCQ) | No | No |
| Rouse,2000(b) | Pre- posttes t, 2 groups  ; RCT | USA | e-learning | 20/26;  Nursing Students | Pediatric nursing | computer- assisted  instruction  +traditional class room lecture | CD-ROM,  computer | >1  seme ster | Absent | Low | Abs ent | No del ay | Objective(MCQ) | No | No |
| Gagnon2013 | Posttes t 2- group; RCT | Canad a | t raditiona l learning | 52/50;  Nursing undergradu ates | Critical reading of scientific articles | Internet-based tutorials +in- class  sessions; | interactive,  Internet-based modules | 1  seme ster | Present(small- group exercises, quizzes) | High(lass discussion, small-group exercises, quizzes.) | Pre sent | No del ay | Objective(MCQ, open-ended questions) | No | No |
| Boynton,2007 | Posttes t 2- group; NRS | USA | t raditiona l learning | 98/107;  Dental students | Pediatric Behavior Managemen t | Internet-based instructional tool+lectures | web-based  instructional tool | 6  week s | Absent | High(essay question) | Pre sent | No del ay | Objective(MCQ, short essay) | No | No |
| LLambi,2011 | Pre- posttes t, 2 groups  ;NRS | Urugu ay | e-learning | 36/30;  Health professional s | Tobacco Cessation Skills | Face-toface + online activitie | Evi Med system | 3  mont hs | Present(cases) | High(cases, wiki-type collaborativ e activity group- discussion workshops) | Pre sent | No del ay | Objective(cannot tell) | No | No |
| Sherman, 2012 | Pre- posttes t, 2 groups  ; RCT | USA | t raditiona l | 35/33;nurses | critical care pharmacolog y | interactive module+discuss ion session | interactive learning modules  delivered via the hospital’s learning management  system | 1  day | Absent | Low | Pre sent | No del ay | Objective(MCQ) | No | No |
| Gerdprasert,201 0 | Pre- posttes t, 2 groups  ; RCT | Thaila nd | t raditiona l | 42/43;nursin g students | mechanism of labour | web-based learning  +conventional lecture | Web-site | 2  week s | Presnt(case scenarios, formative questions and exercises) | High(eb- board for posting questions and discussion between students– students | Pre sent | No del ay | Objective(MCQ. True or false question,  interctive question) | No | No |

|  |  |  |  |  |  |  |  |  |  | and students– teacher) |  |  |  |  |  |
| --- | --- | --- | --- | --- | --- | --- | --- | --- | --- | --- | --- | --- | --- | --- | --- |
| Wahlgren,2006 | Posttes t, 2 groups  ; RCT | Swede n | t raditiona l | 28/85;  medical students | dermatology and  venereology | conventional teaching  +computerised interactsimulat  ion system | computer programming | 17  days | Present(cases,q uexstions) | High(cases, question, extensive feedback) | Pre sent | No del ay | Objective(diagno sis) | No | No |
| **Farrell.2006** | Pre- posttes t, 2 groups  ; RCT | Austra l ia | t raditiona l | 35/41;  nursing students | pharmacolog ical and clinical  contextual  knowledge | Mobile Handheld computers+clini cal practice | Hewlett Packard PDAs (HP iPAQ  Pocket Pc  h5500) | 3  week s | Absent | Low | Abs ent | No del ay | Objective(MCQ) | No | No |
| **Taradi,2004** | Posttes t, 2 groups  ; RCT | *Croati a* | t raditiona l | 37/84;  medical students | acid-base physiology | Online+face-to- face | A  Webenvironme nt created by using the commercially available Web Course Tools (WebCT) | 5  week s | Present(self- testing, exercises, quiz) | High(group collaboratio ns) | Pre sent | No del ay | Objective(MCQ, t rue/false, matching, calculated, short answer, and  written paragraph  questions) | No | No |
| **Kononowicz, 2012** | Pre and Postteses, 2 groups; RCT | *Poland* | traditional | 159 medical students | BSL-AED  Automated external defibrillator | Virtual patient +face-to-face | VP | 6 weeks | Present (questionnaires) | Low | Present | No del ay | Objective(MCQ) | No | No |
| **Lehman, 2015** | Pretestes, 2 groups, RCT | *Australia* | Traditional | 57 medical students | Standard pediatric basic life support | Web-based virtual patient + face-to-face | Web-bases video clip, | 1 years | Present (questionnaires) | Low | Present | 1-2 weeks | Objective(MCQ) | No | No |
| **Succar, 2013** | Pretestes, 2 groups, RCT | *Australia* | Traditional | 188 medical students | Virtual ophthalmology clinic decision | Virtual patient +face-to-face | Conversion navigator | 3-4 years | Present (questionnaires) | Low | Present | 12 monts | Objective(MCQ) | No | No |
| **Wahlgren, 2006** | Pretestes, 2 groups, RCT | *Sweden* | Traditional | 116 medical students | clinical teaching of dermatology and venereology | Virtual patient +face-to-face | NUDOV | 4 year | Present (questionnaires) | Low | Present | No delay | Objective(MCQ) | No | No |
| **Ebadi, 2015** | Pre and Posttestes, 2 groups, RCT | *Iran* | traditional | 30/30 Nurses | Biological incidents | Offline learning, multimedia CD | Lecture presentation | 2 weeks | Absent | Low | Present | No delay | Objective | No | No |
| Liu, 2014 | Pre and Posttestes, 2 groups, RCT | *Taiwan* | traditional | Nurses | Management process | Offline learning | CD-ROM | - | Absent | Low | Present | No delay | Objective | No | No |
| Liu, 2014 | Pre and Posttestes, 2 groups, RCT | *Taiwan* | traditional | Physical therapists | Management process | Offline learning | CD-ROM | 2 weeks | Absent | Low | Present | No delay | Objective | No | No |
| Bayne, 1997 | Pre and Posttestes, 2 groups, RCT | *USA* | traditional | Nurses | Medication and drug overdose | Offline learning | workbook | No delay | Present, questionnaires, 20 items | Low | Present | 4-5 months | Objective | No | No |
| Fernandez-Lao, 2016 | Pre and Posttestes, 2 groups, RCT | *Spain* | traditional | Physiotherapy students | Palpation and ultrasound imaging skills | ODE | Tablet/smartphone | Two weeks | Present, questionnaire, 20 itmes | High (feedback on assignments) | Present | No delay | Objective | No | No |
| Johnston, 2010 | Pre and Posttestes, 2 groups, RCT | *USA* | traditional | Neurosurgery trainees | Neurosurgery-performing ventriculostomy | ODE | Ipod | No delay | Present, 4 items | Hight | Present | No delay | Objective | No | No |
| Kucuk, 2016 | Pre and Posttestes, 2 groups, RCT | *Turkey* | traditional | Medical student | Neutoanatomy | ODE | Tablet/smartphone | No delay | Present | High (feedback on assignments) | Present | No delay | Objective (MCQ) | No | No |
| Ilic, 2015 | Posttestes, 2 groups, RCT | *Australia* | traditional | MBBS program | Ebidence based medicine | online | e-learning | No delay | Present, questionnaire | Low | Present | No delay | Objective (MCQ) | No | No |
| Shimizu, 2019 | Posttestes, 2 groups, RCT | *Japan* | traditional | Internal medicine student | Hematology and endrocrinologt | Online-computer-based test | e-learning | 2 months | Present, questionnaire | Low | Present | No delay | Objective (MCQ) | No | No |
| Milic, 2016 | Posttestes, 2 groups, RCT | Serbia | traditional | Undergraduate medicine students | Final examination | Online-computer-based test | e-learning | 1 year | Present, questionnaire | Low | Present | No delay | Objective (MCQ) | No | No |
| Sadeghi, 2014 | Posttestes, 2 groups, RCT | Iran | traditional | Medical students | Educational contents | Online-computer-based test | e-learning | No delay | Absent | Low | Present | No delay | Objective (MCQ) | No | No |
| Kho, 2018 | Posttestes, 2 groups, RCT | Malaysia | traditional | Medical doctors | Emergency airway management | Online-computer-based test | e-learning | 6 months | Absent | Low | Present | No delay | Objective (MCQ) | No | No |
| Jarrett-Thelwell, 2019 | Posttestes, 2 groups, RCT | *USA* | traditional | Medical doctors | Radiology courses | Online-computer-based test | e-learning | 1 year | Absent | Low | Present | No delay | Objective (MCQ) | No | No |

a. no.(B/N) means number of participants in blended learning versus number of participants in no intervention or non-blended learning.

## E-Table 4. Standard knowledge score and source

|  |  | **Blended** |  |  | **Control** |  |
| --- | --- | --- | --- | --- | --- | --- |
|  | **Total** | **Standard Mean** | **Standard SD** | **Total** | **Standard Mean** | **Standard SD** |
| Ebadi 2015 | 30 | 24,3 | 5,1 | 30 | 13,9 | 3,2 |
| Liu 2014 | 58 | 74 | 12 | 100 | 58 | 12 |
| Liu 2014 | 58 | 91 | 8,6 | 20 | 58 | 20,4 |
| Bayne 197 | 14 | 82,1 | 11,88 | 17 | 81,1 | 13 |
| Fernandez-lao 2016 | 25 | 7,21 | 1,98 | 24 | 8,09 | 0,9 |
| Johnston 2010 | 43 | 80,21 | 5,76 | 12 | 82,75 | 4,69 |
| Kucuk 2016 | 34 | 78,14 | 16,19 | 36 | 68,34 | 12,83 |
| Kulier 2012 | 123 | 69,52 | 5,95 | 81 | 61,45 | 6,2 |
| Kavadella 2012 | 24 | 80,88 | 13,82 | 22 | 68,64 | 13,9 |
| Sowan 2013 | 105 | 78 | 5,5 | 105 | 70 | 8,5 |
| Lancaster 2012 | 29 | 96,6 | 1,9 | 23 | 95,7 | 3,8 |
| Dankbaar 2014 | 31 | 76 | 2 | 16 | 75 | 3 |
| Dankbaar 2014 | 31 | 73 | 2 | 16 | 68 | 3 |
| Mangione 1991 | 13 | 78,5 | 18,28 | 13 | 70 | 22,28 |
| Mangione 1991 | 13 | 78,5 | 18,28 | 9 | 62,5 | 19,85 |
| Shomaker 2002 | 24 | 44,6 | 8,98 | 24 | 51 | 10,14 |
| Shomaker 2002 | 24 | 44,6 | 8,98 | 17 | 51,2 | 10,14 |
| Stewart 2013 | 34 | 75 | 12,25 | 37 | 67,5 | 11,75 |
| Mahnken 2011 | 32 | 72,9 | 12,3 | 32 | 69 | 12,4 |
| Mahnken 2011 | 32 | 87,7 | 12,8 | 32 | 69 | 12,4 |
| Sung 2008 | 24 | 82,21 | 8,75 | 26 | 67,92 | 7,17 |
| Woltering 2009 | 74 | 63,2 | 14,08 | 71 | 55,76 | 12,28 |
| Lowe 2001 | 39 | 31,8 | 15,2 | 46 | 25 | 16,7 |
| Lowe 2001 | 39 | 50,3 | 14 | 46 | 50,2 | 17,4 |
| Hilger 1996 | 45 | 78,4 | 8,98 | 32 | 73,4 | 10,14 |
| Ilic 2013 | 34 | 40,53 | 18 | 27 | 45,13 | 22,4 |
| Morales 2012 | 22 | 72,3 | 6,2 | 22 | 74,2 | 8,1 |
| Raupach 2010 | 40 | 84,8 | 1,3 | 34 | 79,5 | 1,4 |
| Carbonaro 2008 | 22 | 32,44 | 7,33 | 22 | 34 | 10,67 |
| Pereira 2007 | 65 | 63 | 13 | 65 | 50 | 16 |
| Devitt 2001 | 85 | 61,67 | 1,11 | 20 | 45 | 2,5 |
| Kiviniemi 2014 | 38 | 93,92 | 2,45 | 28 | 91,76 | 4,95 |
| Hsu 2011 | 113 | 80,28 | 10,84 | 88 | 81,96 | 10,56 |
| Hsu 2011 | 113 | 66,41 | 8,46 | 88 | 68,11 | 8,73 |
| Kaveevivitchai 2009 | 40 | 61,1 | 6,23 | 40 | 59,43 | 7,83 |
| Kumrow 2005 | 18 | 97,15 | 2,56 | 15 | 84,78 | 3,37 |
| Howerton 2004 | 25 | 84,4 | 9,28 | 24 | 82,5 | 10,07 |
| Howerton 2004 | 25 | 84,4 | 9,28 | 26 | 75 | 7,07 |
| Mars, 1996 | 34 | 65,6 | 8,98 | 34 | 60,7 | 10,14 |
| Gadbury-amyot 2012 | 309 | 95,75 | 10 | 300 | 92 | 12,75 |
| Perkins 2010 | 275 | 84,5 | 11,58 | 276 | 84,92 | 11,5 |
| Strickland 2008 | 8 | 86 | 8,98 | 6 | 85 | 10,14 |
| Rouse 2000 | 20 | 77,3 | 11,5 | 26 | 66,2 | 11,6 |
| Rouse 2000 | 20 | 77,3 | 11,5 | 26 | 74 | 11 |
| Gagnon 2013 | 52 | 17,2 | 0,9 | 50 | 14,5 | 0,6 |
| Boynton 2007 | 98 | 78,22 | 7,67 | 107 | 74,72 | 12,56 |
| Llambi 2011 | 36 | 83,1 | 2,8 | 30 | 75,3 | 17,2 |
| Sherman 2012 | 35 | 89,7 | 5,16 | 33 | 88,3 | 6,79 |
| Gerdpraset 2010 | 42 | 71,9 | 9,59 | 43 | 87,93 | 5,76 |
| Farrell 2006 | 35 | 50,66 | 8,98 | 41 | 45,34 | 10,14 |
| Taradi 2004 | 37 | 71,69 | 1,83 | 84 | 61,33 | 1,03 |
| Kononowicz 2012 | 51 | 47,37 | 3,43 | 45 | 48,81 | 3,76 |
| Lehman 2015 | 27 | 92,2 | 4,7 | 30 | 68,8 | 16,3 |
| Succar 2013 | 76 | 16 | 1,8 | 74 | 14,8 | 2,2 |
| Wahlgren 2006 | 28 | 88,8 | 9,38 | 85 | 87,5 | 10 |
| Ilic 2015 | 73 | 40,53 | 18 | 74 | 45,13 | 22,4 |
| Shimizu 2019 | 72 | 58 | 8 | 24 | 56,6 | 10,2 |
| Milic 2016 | 108 | 7,88 | 1,3 | 437 | 7,51 | 1,36 |
| Sadeghi 2014 | 48 | 16,81 | 1,06 | 45 | 16,51 | 0,69 |
| Kho 2018 | 15 | 35 | 1,75 | 15 | 31 | 1,81 |
| Jarrett-Thewell 2019 | 178 | 91,3 | 4,8 | 184 | 90 | 6,04 |
| Zhan 2017 | 464 | 93,61 | 0,61 | 476 | 88,29 | 0,75 |
| Zhan 2017 | 474 | 94,05 | 0,59 | 479 | 90,22 | 0,53 |
| Zhan 2017 | 445 | 93,88 | 0,64 | 485 | 89,09 | 0,78 |
| Noll 2017 | 22 | 7 | 1,48 | 22 | 7,77 | 1,51 |
| Noll 2017 | 22 | 6,67 | 1,62 | 22 | 6,63 | 1,3 |
| McCutcheon 2018 | 56 | 4,2 | 1,43 | 57 | 3,51 | 1,51 |
| Marchalot 2018 | 54 | 232 | 18,75 | 95 | 215 | 27,35 |

# E-table 5. PRISMA Checklist

| **Section/topic** | **#** | **Checklist item** | **Reported on** | |
| --- | --- | --- | --- | --- |
|  |  |  | **heading** | |
| **T ITLE** |  |  |  | |
| on | 1 | Effectiveness of à blended learning course and flipped classroom in first year anaesthesia training | Title (page 1) | |
| **ABSTRACT** | | |  |  |
| Structured summary | 2 | Provide a structured summary including, as applicable: background; methods; data sources; study eligibility criteria, participants, and interventions; study appraisal and synthesis methods; results; limitations; conclusions and implications of key findings; systematic review registration number. | Abstract (page 1) | |
| **I N TRODUCT ION** | | |  |  |
| Rationale | 3 | Describe the rationale for the review in the context of what is already known. | Introduction (page 1- 2 ) | |
| Objectives | 4 | Provide an explicit statement of questions being addressed with reference to participants, interventions, comparisons, outcomes, and study design (PICOS). | Introduction (page 2) | |
|  |  |  |  | |
| **MET HODS** | | |  |  |
| Protocol and registration | 5 | Indicate if a review protocol exists, if and where i t can be accessed (e.g., Web address), and, if available, provide registration information including registration number. | N/A | |
| Eligibility criteria | 6 | Specify study characteristics (e.g., PICOS, length of follow-up) and report characteristics (e.g., years considered, language, publication status) used as criteria for eligibility, giving rationale. | Methods: Eligibility criteria (page 2-3) | |
| Information sources | 7 | Describe all information sources (e.g., databases with dates of coverage, contact with study authors to identify additional studies) in the search and date last searched. | Methods: Data sources (page 2 -3) | |
| Search | 8 | Present full electronic search strategy for at least one database, including any limits used, such that i t could be repeated. | Methods: (page 2) E-Link | |
|  |  |  |  | |
| Study selection | 9 | State the process for selecting studies (i.e., screening, eligibility, included in systematic review, and, if applicable, included in the meta-analysis). | Methods: Resident selection (page 2) | |
|  |  |  |  | |
| Data collection process | 10 | Describe method of data extraction from reports (e.g., piloted forms, independently, in duplicate) and any processes for obtaining and confirming data from investigators. | Methods: Data extraction (page 2 )3) | |
| **Section/topic** | **#** | **Checklist item** | **Reported on** | |
|  |  |  | **Heading** | |
| **MET HODS (cont.)** | | |  |  |
| Data i tems | 11 | List and define all variables for which data were sought (e.g., PICOS, funding sources) and any assumptions and simplifications made. | Methods: Data extraction (page 2 - 3) | |
| Risk of bias in individual studies | 12 | Describe methods used for assessing r isk of bias of individual studies (including specification of whether this was done at the study or outcome level), and how this information is to be used in any data synthesis. | Methods: in Discussion, page 5 | |
|  |  |  |  | |
|  |  |  |  | |
| Summary measures | 13 | State the principal summary measures (e.g., r isk ratio, difference in means). | Methods: outcomes variables (page 3) | |
| Synthesis of results | 14 | Describe the methods of handling data and combining results of studies, if done, including measures of consistency (e.g., I 2) for each meta-analysis. | Methods: outcome variable (page 3) | |
| Risk of bias across studies | 15 | Specify any assessment of r isk of bias that may affect the cumulative evidence (e.g., publication bias, selective reporting within studies). | N/À | |
| Additional analyses | 16 | Describe methods of additional analyses (e.g., sensitivity or subgroup analyses, meta-regression), if done, indicating which were pre-specified. ) | N/À | |
| **RESULTS** |  |  |  | |
| Study selection | 17 | Give numbers of studies screened, assessed for eligibility, and included in the review, with reasons for | Results: partcipants, primary outcome, secondary outcomes (page 3) | |
|  |  | exclusions at each stage, ideally with a f low diagram. | | |
|  |  |  |  | |
|  |  |  |  | |
| Study characteristics | 18 | For each study, present characteristics for which data were extracted (e.g., study size, PICOS, follow- up period) and provide the citations. | Results: Table 1 (page 4) | |
|  |  |  |  | |
|  |  |  |  | |
| Risk of bias within studies | 19 | Present data on r isk of bias of each study and, if available, any outcome level assessment (see i tem 12). | N/À | |
| Results of individual | 20 | For all outcomes considered (benefits or harms), present, for each study: (a) simple summary data for | N/À | |
| studies |  | each intervention group (b) effect estimates and confidence intervals, ideally with a forest plot. | | |
|  |  |  |  | |
|  |  |  |  | |
|  |  |  |  | |
| Synthesis of results | 21 | Present results of each meta-analysis done, including confidence intervals and measures of consistency. | Results: | |
|  |  |  | Figure 2 (page | |
|  |  |  | 14) and Figure 4 (page 18) | |
| **Section/topic** | **#** | **Checklist item** | **Reported on** | |
|  |  |  | **Heading** | |
| **RESULTS (cont.)** | | |  |  |
| Risk of bias across studies | 22 | Present results of any assessment of r isk of bias across studies (see I tem 15). | | |
|  |  |  | N/À | |
|  |  |  |  | |
| Additional analysis | 23 | Give results of additional analyses, if done (e.g., sensitivity or subgroup analyses, meta-regression [see I tem 16]). | Results: Figure 2 (page 4) | |
|  |  |  |  | |
|  |  |  |  | |
| **D ISCUSSION** | | |  |  |
| Summary of evidence | 24 | Summarize the main findings including the strength of evidence for each main outcome; consider their relevance to key groups (e.g., healthcare providers, users, and policy makers). | Discussion (page 4) | |
| Limitations | 25 | Discuss limitations at study and outcome level (e.g., r isk of bias), and at review-level (e.g., incomplete retrieval of identified research, reporting bias). | Discussion (pager 5) | |
| Conclusions | 26 | Provide a general interpretation of the results in the context of other evidence, and implications for future research. | Conclusion (page 5) | |
| **FUND ING** |  |  |  | |
| Funding | 27 | Describe sources of funding for the systematic review and other support (e.g., supply of data); role of funders for the systematic review. | Acknowledgem ents (page 5) | |

***From*** *Marchalot, A., Dureuil, B., Veber, B., Fellahi, J.-L., Hanouz, J.-L., Dupont, H., et al. (2018). Effectiveness of a blended learning course and flipped classroom in first year anaesthesia training. Anaesth. Crit. Care Pain Med. 37, 411–415. doi:10.1016/j.accpm.2017.10.008.*
